# Genome-Wide Methylation Sequencing to Identify DNA Methylation Markers for Early-stage Hepatocellular Carcinoma in Liver and Blood

**DOI:** 10.1186/s13046-025-03412-9

**Published:** 2025-05-15

**Authors:** Siyu Fu, Ruben G. Boers, Joachim B. Boers, Pam E. van der Meeren, Jean Helmijr, Vanja de Weerd, Michail Doukas, Maurice Jansen, Bettina E. Hansen, Roeland F. de Wilde, Dave Sprengers, Joost Gribnau, Saskia M. Wilting, José D. Debes, Andre Boonstra

**Affiliations:** 1 Department of Gastroenterology and Hepatology, Erasmus University Medical Center, Wytemaweg 80, 3015 CN Rotterdam, The Netherlands; 2 Department of Developmental Biology, Erasmus University Medical Center, Rotterdam, The Netherlands; 3 Department of Surgery, Erasmus MC Transplant Institute, Erasmus University Medical Center, Rotterdam, The Netherlands; 4 Department of Medical Oncology, Erasmus University Medical Center, Rotterdam, The Netherlands; 5 Department of Pathology, Erasmus University Medical Center, Rotterdam, The Netherlands; 6 Toronto Centre for Liver Disease, University Health Network, University of Toronto, Toronto, Canada; 7 Department of Epidemiology, Biostatistics, Erasmus University Medical Center, Rotterdam, the Netherlands; 8 Department of Medicine, University of Minnesota, Minneapolis, MN 55455 USA

**Keywords:** Hepatocellular carcinoma, DNA methylation markers, Cell-free DNA, Methylated DNA sequencing

## Abstract

**Background:**

Hepatocellular carcinoma (HCC) is associated with a poor 5-year survival mainly due to detection at late stages. Better non-invasive surveillance methods are needed to improve early detection and maximize survival. We performed a strict assessment of DNA methylation markers (DMMs) for HCC detection.

**Methods:**

A total of 385 samples from liver tissues and blood were analyzed. Genome-wide Methylated DNA sequencing (MeD-seq) was initially performed on 46 liver tissues, followed by the validation using quantitative methylation-specific PCR (qMSP) on 175 liver tissues. The selected DMMs with and without ASAP/GAAD score were further evaluated in 180 blood samples. Additionally, MeD-seq was performed to validate the results on blood.

**Results:**

MeD-seq revealed a substantial number of differentially methylated regions (DMRs) in HCC tissues compared to non-HCC controls. By qMSP, the top 5 DMMs demonstrated strong performance in distinguishing cirrhotic HCC from cirrhosis controls in tissue (AUC 0.842 to 0.957). However, evaluation of these DMMs in blood showed lower performance in early HCC detection compared to cirrhosis in both the training (sensitivity 26.7–43.3%, 81.3% specificity) and validation cohorts (sensitivity 16.2–43.2%, 85.7% specificity). The addition of DMMs to the ASAP/GAAD score only provided an additional 5.4% sensitivity in the validation cohort compared to the ASAP/GAAD score alone. These findings were confirmed using MeD-seq analysis in blood samples, which revealed no detectable DMRs between cirrhotic HCC and cirrhosis controls. Interestingly, DNA methylation patterns in blood of healthy individuals differed strongly from both groups (cirrhosis and cirrhotic HCC).

**Conclusion:**

DNA methylation patterns in liver tissue were distinctly different between HCC and controls. In blood, DMMs contributed minimally to early-stage HCC detection compared to cirrhosis, whether used alone or in combination with the ASAP/GAAD score. It is likely that high baseline DNA methylation related to cirrhosis and possibly the low input of tumor-related DNA impacts the use of DMMs in early HCC detection in blood.

**Supplementary Information:**

The online version contains supplementary material available at 10.1186/s13046-025-03412-9.

## Background

Worldwide, liver cancer is a leading cause of cancer-related mortality. In 2020, approximately 900,000 new diagnoses of liver cancer and 800,000 deaths were reported [1]. Hepatocellular carcinoma (HCC) constitutes over 90% of primary liver cancers. The development of HCC often occurs in the background of chronic liver disease, particularly among patients with advanced fibrosis or cirrhosis [2]. Risk factors contributing to HCC include alcohol-related liver disease (ALD), viral hepatitis and metabolic dysfunction-associated steatotic liver disease (MASLD) [3, 4]. Early detection of HCC is crucial for optimal outcome, as in more advanced disease stage, curative options are less abundant [5–7].

In clinical practice, ultrasound is widely used for the surveillance and detection of HCC in high-risk individuals, but its effectiveness in early detection is significantly limited considering a sensitivity of only 47% [8]. Alpha-fetoprotein (AFP) is currently the only biomarker utilized for the detection and monitoring of HCC [5], but AFP testing has a sensitivity of only 39–64% for early-stage HCC [9]. Moreover, AFP contributes to identifying only an additional 6–8% of cases not detected by ultrasound [7, 10]. Efforts to improve the early detection of HCC have led to the development of algorithms for HCC risk score, such as the GALAD score [11–13] and the ASAP/GAAD score [14, 15]. Although these scores demonstrated improved performance in detecting early HCC, AFP is still a major contributor to the scores, and without optimal AFP sensitivity, their efficacy in identifying early HCC remains suboptimal. Therefore, additional non-invasive biomarkers to further improve early detection of HCC are needed.

The potential use of DNA methylation markers (DMMs) as novel biomarkers is gaining increasing attention and several DMMs have already been identified for different tumor types [16]. Altered DNA methylation profiles are a hallmark of cancer, and numerous studies have demonstrated that tumor methylation profiles differ from the non-tumor tissue [17–19]. Detection of DNA methylation in blood is facilitated by the presence of cell-free DNA (cfDNA) circulating in the bloodstream [20]. This cfDNA is released into circulation through various physiological and pathological cellular processes, including cellular turnover, apoptosis, and necrosis [21]. In this regard, our team has previously demonstrated the utility of DMMs in the detection, prognosis and treatment response evaluation using cfDNA of patients with colorectal cancer [22], ovarian cancer [23], and renal cell carcinoma [24]. Also, in livers of HCC patients, DNA methylation changes have been shown to be strongly associated with the development and progression of HCC [25, 26], and several studies have reported aberrant DNA methylation events in cfDNA of HCC [27, 28]. However, an important difference with other types of cancers, such as colon and renal cancer, is that the majority of HCC cases develop on a background of cirrhosis, which may lead to elevated DNA methylation levels [29]. Although previous studies have shown that levels of DMMs increased in HCC samples, these findings lack robust validation. In addition, to date, most DMMs have been identified through the sequencing of HCC liver tissues, leaving it unclear whether more sensitive DMMs can be found based on the sequencing of blood. One of the reasons for this is the low amount of cfDNA that can be obtained from blood, which is insufficient for sequencing using some previously reported DNA methylation techniques.

To address these questions and perform a comprehensive assessment of DNA methylation changes in both liver tissues and blood, we conducted an extensive evaluation of DMMs in well-designed patient cohorts. By using the previously developed DNA methylation sequencing technique, Methylated DNA sequencing (MeD-seq), which covers over 50% of all potentially methylated CpGs across the genome [30], we analyzed genome wide DNA methylation patterns using MeD-seq and selected regions using quantitative methylation-specific PCR (qMSP) in both liver tissue and blood. Our goal is to evaluate the feasibility of implementing DMMs for the early detection of cirrhotic HCC in future clinical practice.

## Methods

### Patient samples

Snap-frozen liver tissue and plasma from patients were selected from biobanks of the departments of Pathology or Gastroenterology and Hepatology of the Erasmus MC. For the study on DNA methylation levels in liver tissue, 27 cirrhotic HCC, 54 non-cirrhotic HCC, 44 cirrhotic non-HCC, 36 non-cirrhotic non-HCC, and 14 benign hepatic lesions were selected. The tissue was sampled at the time of surgical resection or was taken as a biopsy prior to systemic chemotherapy or local/regional therapy. Patients with sufficient clinicopathological information about the etiology (i.e. viral, non-viral, or cryptogenic) of the liver disease and their cirrhosis status were included in the study. Patients were excluded in case of co-existing non-HCC malignancy, recurrent HCC, or were younger than 18 years.

In all cases, characterization of HCC patients was based on imaging and/or histological evidence, in accordance with HCC guidelines [7], and selected HE-stained liver tissue samples were all re-assessed by an expert liver pathologist to confirm pathological diagnoses and evaluate the percentage of tumor-related available tissue. HCC samples exhibited a tumor percentage greater than 70% in 75 of 81 cases.

Blood samples from 122 HCC patients with cirrhosis and 60 non-HCC cirrhotic controls were obtained from the ESCALON Horizon2020 cohort (www.escalon.eu), collected since 2019. In addition, blood of 10 healthy individuals was obtained from the Dutch National blood bank (Sanquin) [22]. For the paired evaluation of DNA methylation levels in tissue and plasma, samples from 9 patients were collected. Data similar to the ones provided for the tissue samples were included. At the time of blood collection, HCC patients had not received prior surgical treatment or chemotherapy. Exclusion criteria were the same as those applied in tissue samples. Additional exclusion criteria for blood samples were current use of vitamin K inhibitors (due to the impact on PIVKA-II serum levels), current pregnancy (could affect serum AFP levels), and/or age < 18 years. To confirm the absence of HCC in cirrhosis controls, a minimum follow-up of 12 months (80% of the samples had follow-up periods of more than 2 years) after blood collection was required. Serum and plasma samples were processed from peripheral blood within 4 h after drawing and stored at −80 °C.

### Determination of clinical parameters

Electronic medical records related to imaging, pathology, and laboratory tests were used to assess clinical information. HBV or HCV infections status was diagnosed serologically. Patients with persistent ethanol intake of more than 40 g/day for men and 30 g/day for women for over 10 years, without other liver damage triggers were assigned as alcohol-associated liver disease (ALD). MASLD patients were diagnosed by the managing hepatologists or evidence of hepatic steatosis observed through ultrasound or histopathology, in the absence of other liver diseases. Patients in whom other etiologies (hemochromatosis, primary biliary cholangitis, primary sclerosing cholangitis, a1-antitrypsin deficiency, and Wilson's disease) of liver disease were present, were excluded and assigned to cryptogenic. The Barcelona Clinic Liver Cancer (BCLC) staging system was used to assign the tumor stage in case cirrhosis was present [6], classifying the disease to early-stage (BCLC stage 0 and A) and late-stage (BCLC B-D). The state of cirrhosis was determined through pathology (Metavir F4) or liver transient elastography (> 12.0 kPa).

Levels of AFP and PIVKA-II were determined in 180 serum samples from patients with cirrhotic HCC (*n* = 120) and cirrhotic controls (*n* = 60) with the LUMIPULSE G AFP-N and PIVKA-II kit using the LUMIPULSE G1200 instrument (Fujirebio) in accordance with manufacturer’s instructions. Low and high limits of detection were for AFP 0.5 and 2000 ng/ml, and for PIVKA-II 5 and 75,000 mAU/ml. The algorithm of ASAP score was described before [14]: Z = − 6.836 + 0.042 × age + 0.989 × gender (1 for male, 0 for female) + 1.841 × log10 (AFP) + 0.949 × log10 (PIVKA-II).

### DNA isolation and quantification

Genomic DNA was isolated from snap-frozen liver tissues using the QIAamp DNA Mini Kit (Qiagen) according to the manufacturer’s instructions. DNA was eluted in AE elution (10 mM Tris·Cl; 0.5 mM EDTA; pH 9.0), and the concentration was quantified with NanoDrop (ThermoFisher). cfDNA was extracted from 1–2 ml of plasma using the QIAamp circulating nucleic acid kit (Qiagen) according to the manufacturer’s instructions. cfDNA was eluted in AVE buffer (RNase-free water with 0.04% NaN_3_). The concentration of cfDNA was measured on a Qubit 2.0 Fluorometer (Invitrogen). DNA samples were stored at − 80 °C for later analysis.

### Methylated DNA sequencing (MeD-seq)

MeD-seq assays were performed as previously described [30]. In short, 90 ng of isolated genomic DNA from liver tissues and 10 ng of cfDNA from plasma samples were digested using LpnPI enzymes (New England Biolabs), generating 32 basepair (bp) fragments around the methylated recognition site containing a CpG. The digested DNA fragments were further processed using the ThruPlex DNA-seq 96D kit (Rubicon Genomics). Stem-loop adapters were blunt-end ligated to repaired input DNA, then amplified to include dual-indexed barcodes using a high-fidelity polymerase to generate an indexed Illumina NGS library. The amplified end product was purified on a Pippin HT system with 3% agarose gel cassettes (Sage Science). Libraries were multiplexed and sequenced on an Illumina NextSeq2000 system for paired-end reads of 50 bp according to the manufacturer's instructions. The dual-indexed samples were de-multiplexed using bcl2fastq v2.20 software (Illumina). Samples with over 20 million total reads and more than 20% of CpGs relative to the total reads are considered of good quality and are processed for analysis.

### Processing of MeD-seq data

MeD-seq data was processed and analyzed with Python 3.9.11 using customized scripts as described previously [30]. Briefly, the raw FASTQ files were subjected to Illumina adaptor trimming and filtered for the presence of LpnPI restriction sites 13–17 bp from the 3’ or 5’ end. Next, reads were mapped to the Hg38 genome using bowtie 2.1.0, BAM files were generated using SAMtools and visualized using IGV 2.11.2 (Broad Institute). LpnPI site scores were used to produce read count scores for the transcription start sites (TSS; 1 kb before and 1 kb after), gene bodies (1 kb after the TSS until the transcription end-site) and CpG islands, based on reference annotations from the UCSC database (hg38). To detect differentially methylated regions (DMRs), a genome-wide sliding window was used to detect sequentially differentially methylated LpnPI sites between two groups of the derivation cohort, genome-wide read counts were normalized (RPM, reads per million) for coverage and compared using the Chi-Square test, with significance set at *p* < 0.05 and a Bonferroni correction for multiple testing. Neighboring significantly called LpnPI sites were binned and reported. Overlap of genome-wide detected DMRs was reported for TSS, CpG islands or gene bodies regions using the annotations of the UCSC database (hg38). DMR thresholds were based on LpnPI site count, DMR sizes (in bp) and fold changes of read counts before performing clustering.

In order to determine the presence or absence of group-associated DNA methylation signatures per sample, receiver operating characteristic (ROC) curves were calculated for each individual DMR. ROC curves between groups were used to calculate the optimal threshold (using the “scikit-learn” package Python) for each individual DMR. Samples above the threshold scored 1, samples under the threshold scored 0. A cumulative score was generated for all DMRs resulting in DNA methylation scores associated with each group. DNA methylation scores were then compared between groups using DESeq2 analysis, focusing on fold-change with a false discovery rate (FDR) of less than 0.01.

### Quantitative methylation-specific PCR

The DNA methylation status of the selected DMMs was determined by quantitative methylation-specific PCR (qMSP) assay on bisulfite-converted genomic DNA of liver tissues and cfDNA of plasma samples. Briefly, 100 ng genomic DNA and 5 ng cfDNA were modified via the EZ DNA Methylation kit (Zymo Research). For the paired tissue–plasma samples, 10 ng of DNA from each sample was used. The modified DNA was used for each DNA methylation analysis using the EpiTect MethyLight Master Mix (Qiagen). Primers and probes were designed to amplify the methylated DNA sequence, and quantify the amplicons, respectively (Supplementary Table 1; Eurogentec). Specificity of methylation primers was confirmed with the EpiTect PCR Control DNA Set (Qiagen), which contains bisulfite-converted methylated and unmethylated DNA, and unconverted unmethylated DNA. All PCR reactions were positive for bisulfite-converted methylated DNA, while negative for the controls. The qMSP reaction was conducted in a 12.5 µL reaction volume containing 6.25 µL 2 × EpiTect MethyLight Master Mix (w/o ROX), 2 µL bisulfite-converted DNA, 400 nM per primer, and 200 nM probe using the StepOnePlus or QuantStudio 3 Real-Time PCR System (ThermoFisher). The modified and unmethylated sequence of β-actin (*ACTB*) was amplified as a reference [31]. Methylation levels were normalized by *ACTB* using the comparative Ct method (2 − ΔCT) [32].

### Statistics

Statistical analyses were performed using SPSS 28.0.1.0. Continuous variables were presented as medians (interquartile ranges) and categorical variables as percentages. Descriptive statistics were used to summarize the patient characteristics in the case and control groups. The Chi-Square test was used for testing dichotomous variables and the Mann–Whitney U test for continuous variables. Wilcoxon signed-rank test was used to compare DNA methylation levels between paired plasma and tissue samples. Binary logistic regression (Enter or Backward elimination) was used to develop an algorithm consisting of the selected DMMs and the ASAP/GAAD score. The area under the curve (AUC) were used to determine the performance of the following biomarkers: DMMs, AFP, PIVKA-II, ASAP/GAAD and the combination between ASAP/GAAD and DMMs. The Akaike Information Criterion (AIC) was calculated to assess both the complexity and effectiveness of the models. The performance of the ASAP/GAAD score was compared with that of individual DMMs and the combination of DMMs at a fixed specificity. A two-tailed value of *p* < 0.05 was considered statistically significant.

Principal component analysis (PCA) was performed on the DMRs between cirrhotic HCC and cirrhosis tissues to reduce the dimensionality of the data. The normalized data were mean-centered and reduced to two principal components using R studio 4.1.1.

## Results

### In liver tissue, MeD-seq demonstrated differential DNA methylation patterns in HCC patients compared to controls

To conduct a comprehensive investigation into DNA methylation patterns in HCC liver tissue, we initially performed MeD-seq on DNA of 46 frozen tissue samples from patients with ALD to explore HCC-specific DNA methylation patterns in livers from patients with cirrhotic HCC (*n* = 6), cirrhosis (*n* = 15), non-cirrhotic HCC (*n* = 8), non-cirrhotic liver disease (*n* = 10), and benign hepatic lesions (*n* = 7), as detailed in Table 1.

**Table 1:** Clinical parameters for MeD-seq on ALD-related liver disease

	Liver tissue (*n* = 46)					cfDNA (*n* = 42)			
Group	Cirrhotic HCC	Cirrhosis	Non-cirrhotic HCC	Non-cirrhotic liver disease	*Benign hepatic lesions	Late-stage HCC	Early-stage HCC	Cirrhosis	Healthy individuals
N	6	15	8	10	7	10	10	12	10
Age, median	67	54	67	47	46	71	71	65	52
Gender, male	5 (83.3%)	10 (66.7%)	5 (62.5%)	7 (70.0%)	1 (14.3%)	9 (90.0%)	9 (90.0%)	12 (100%)	5 (50.0%)
Cirrhosis	100%	100%	0%	0%	0%	100%	100%	100%	0%
BCLC-Stage									
Early-stage/BCLC 0-A	4 (66.7%)	NA	4 (50.0%)	NA	NA	0	10 (100%)	NA	NA
Intermediate/BCLC B	2 (33.3%)	NA	4(50.0%)	NA	NA	8(80.0%)	0	NA	NA
Late-stage/BCLC C-D	0	NA	0	NA	NA	2 (20.0%)	0	NA	NA

*Abbreviations*: *MeD-seq* Methylated DNA sequencing, *HCC* hepatocellular carcinoma, *ALD* alcohol-related liver disease, *BCLC* Barcelona Clinic Liver Cancer staging system, *NA* not available.*3 focal nodular hyperplasia and 4 hepatocellular adenoma

**Fig. 1:**
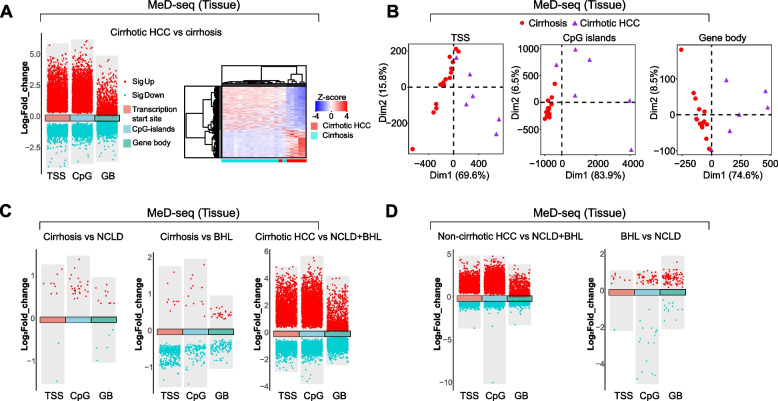
Comparison of DNA methylation patterns in liver tissues using MeD-seq. The volcano plot depicted DNA methylation scores for statistically significant (DESeq2 analysis, FDR < 0.01) hypermethylated (red) and hypomethylated (cyan) DMRs across transcriptional start site (TSS), CpG islands (CpG), and gene bodies (GB) regions. **A** In liver tissues, cirrhotic HCC (*n* = 6) exhibited distinct DNA methylation patterns compared to cirrhotic controls (*n* = 15), demonstrated through both differentially methylation analysis on either the three mentioned regions (TSS, CpG islands and gene bodies) and the full genome-wide analysis which also contains intergenic DMRs (heatmap). **B** Principal component analysis on DMRs of the three mentioned regions (TSS, CpG islands and gene bodies) revealed DNA methylation profiles for each liver sample of cirrhotic HCC and cirrhosis. **C** The comparison of DNA methylation between livers from patients with cirrhosis and non-cirrhotic liver disease (NCLD, *n* = 10) or benign hepatic lesions (BHL, *n* = 7) yielded significantly fewer DMRs than the comparison between liver samples from cirrhotic HCC and non-cirrhotic controls. **D** The comparison of DNA methylation from livers of non-cirrhotic HCC patients (*n* = 8) and non-cirrhotic controls (*n* = 17) exhibited a higher number of DMRs than the comparison between BHL and NCLD. DMRs located on the X- and Y-chromosome were removed to avoid gender-related effects

The MeD-seq methodology enabled the identification of DMRs by calculating the DNA methylation scores for three genomic regions: the transcription start-site (TSS), CpG islands, and gene bodies as well as for the small inter- and intragenic DMRs found using a genome wide sliding window approach. As shown in Fig. 1A, MeD-seq revealed distinct methylation patterns in DNA from cirrhotic HCC liver tissues compared to cirrhotic control livers, identifying 3842 DMRs (either hyper- and hypomethylated) in the TSS, 4417 in CpG islands, and 5154 in the gene bodies, along with a total of 9876 DMRs throughout the genome (heatmap).

As shown in Fig. 1B, principal component analysis of the DMRs (3842 in TSS, 4417 in CpG islands, and 5154 in the gene bodies) exhibited heterogenous DNA methylation patterns for cirrhotic HCC liver samples, whereas the profile for cirrhotic liver controls across the three genomic regions was more clustered, particularly within CpG islands regions.

Importantly, the DNA methylation patterns in cirrhotic liver tissue were similar to those of non-cirrhotic liver disease (NCLD) or benign hepatic lesions (BHL). since only few DMRs were identified (Fig. 1C), indicating that the presence of HCC, and not of cirrhosis, is the determining factor that results in altered DNA methylation in liver tissue. In line with this, MeD-seq analysis of DNA from livers of patients with non-cirrhotic HCC displayed 2140 DMRs in TSS, 3722 in CpG islands, and 2381 in gene bodies compared to non-cirrhotic control livers (Fig. 1D). These findings indicate that DNA methylation changes occur more frequently in livers of cirrhotic as well as non-cirrhotic HCC patients than in control livers of a comparable background, and is closely associated with its carcinogenesis.

### Tissue-derived DMMs from MeD-seq exhibited strong performance in distinguishing cirrhotic HCC from cirrhosis tissues

**Fig. 2:**
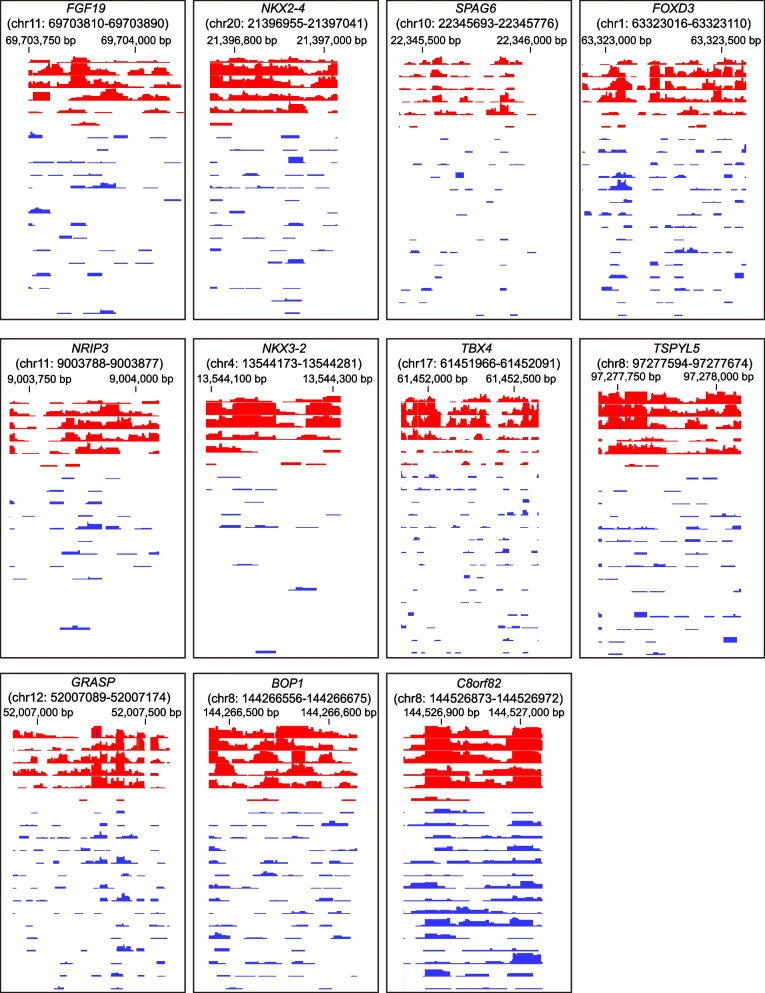
Selection of DMRs from MeD-seq results using IGV software. MeD-seq was conducted on DNA from cirrhotic HCC (red, *n* = 6) and cirrhotic (blue, *n* = 15) liver tissues. The DNA methylation profiles of selected genes, as illustrated by IGV software, are displayed. Quantitative methylation specific PCR (qMSP) primers and probes are designed and targeted on these specific hypermethylated regions for the 11 DMMs

**Fig. 3:**
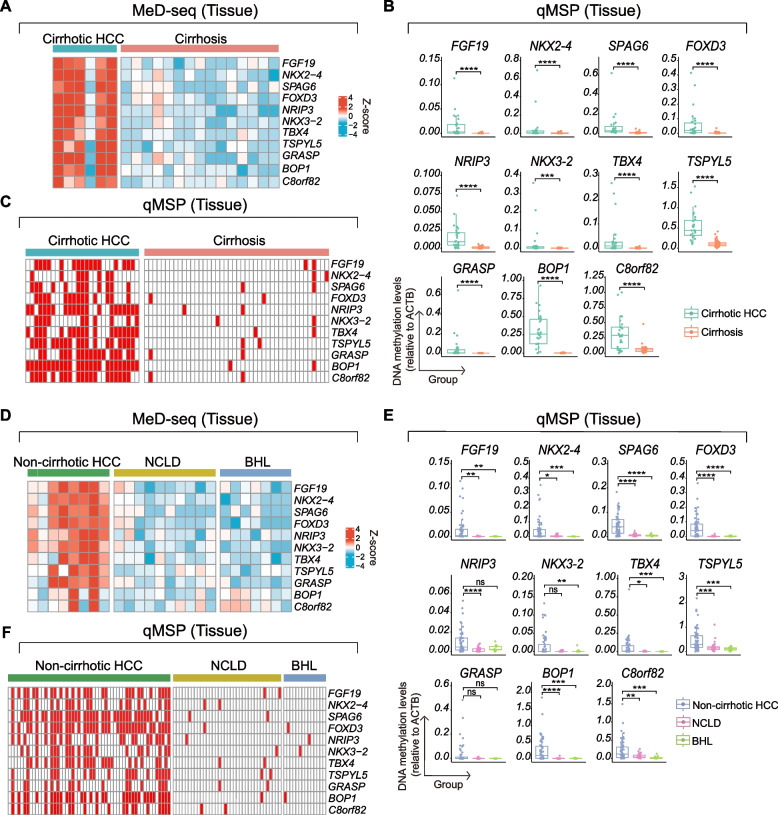
qMSP analysis on DNA from 175 liver tissues. **A** DNA methylation score for the 11 selected DMMs in cirrhotic HCC livers compared to cirrhotic livers. **B** qMSP-based methylation levels relative to the *ACTB* reference gene were shown for the selected DMMs in 27 patients with cirrhotic HCC compared to 44 with cirrhosis (Mann–Whitney U test; ***, *p* < 0.001; ****, *p* < 0.0001). **C** Methylation levels for the 11 DMMs were presented in red or white. The red color indicated methylation values that exceed the 95 th percentile of the control group (cirrhosis) for each DMM (rows) across the tissue samples (columns). White boxes represented values that fall below the 95 th percentile threshold established in the control group. **D** DNA methylation scores of 11 tissue-derived DMMs in samples from non-cirrhotic HCC compared to those from patients with non-cirrhotic liver disease (NCLD) and benign hepatic lesions (BHL). **E** DNA methylation levels of the 11 DMMs, normalized by *ACTB*, were measured using qMSP in 54 non-cirrhotic HCC, 36 NCLD, and 14 BHL tissues (Mann–Whitney U test; ns, not significant; *, *p* < 0.05; **, *p* < 0.01; ***, *p* < 0.001; ****, *p* < 0.0001). **F** qMSP-based DNA methylation levels were represented using different colors, with red indicating values above the 95 th percentile of the control group (NCLD and BHL) and white representing values below this threshold

To confirm the findings from MeD-seq in a larger and more heterogenous cohort of DNA from frozen liver samples, we selected 11 DMMs for further investigation. The criteria for selecting these tissue-derived DMM included a false discovery rate (FDR) of less than 0.01 based on DNA methylation score, and hypermethylation in cirrhotic HCC livers as compared to cirrhotic control livers, as visualized by IGV software (Fig. 2). The selected DMMs were *FGF19*, *NKX2-4*, *SPAG6*, *FOXD3*, *NRIP3*, *NKX3-2*, *TBX4*, *TSPYL5*, *GRASP*, *BOP1*, and *C8orf82*. DNA methylation scores for the 11 DMMs that were significantly higher in 5 out of 6 cirrhotic HCC liver tissues compared to cirrhotic controls (Fig. 3A). Notably, one cirrhotic HCC patient displayed much lower hypermethylation for any of the 11 DMMs, which may be attributable to tumor heterogeneity. Subsequently, the 11 candidate DMMs were tested by qMSP on DNA of 27 cirrhotic HCC liver tissues and 44 cirrhosis tissues (Supplementary Table 2). All 11 DMMs exhibited hypermethylation in cirrhotic HCC livers compared to cirrhotic livers (*p* < 0.05) (Fig. 3B). At the 95 th percentile threshold of the cirrhosis group, significant hypermethylation was observed in 16 out of 27 cirrhotic HCC tissues for *FGF19* (59.3%), 8 for *NKX2-4* (29.6%), 12 for *SPAG6* (44.4%), 14 for *FOXD3* (51.9%), 20 for *NRIP3* (74.1%), 10 for *NKX3-2* (37.0%), 18 for *TBX4* (66.7%), 19 for *TSPYL5* (70.4%), 17 for *GRASP* (63.0%), 25 for *BOP1* (92.6%) and 19 for *C8orf82* (70.4%) (Fig. 3C). These qMSP findings therefore validate the MeD-seq data in a larger, more diverse set of patients with cirrhotic HCC. In a subgroup analysis, we examined the differences between viral and non-viral related etiologies and observed significantly increased DNA methylation levels of *SPAG6* and *NKX2-4* in viral-related (HBV + HCV) cirrhotic HCC compared to non-viral-related (MASLD + ALD) cirrhotic HCC (Supplementary Fig. 1).

Next, we set out to determine whether qMSP could also validate the MeD-seq findings that demonstrated distinctive DNA methylation patterns in liver tissue from non-cirrhotic HCC patients as compared to non-cirrhotic control livers. Indeed, qMSP on the original samples run by MeD-seq revealed that 6 of 8 samples (75%) showed hypermethylation in almost 9 out of 11 DMMs in non-cirrhotic HCC compared to non-cirrhotic controls (Fig. 3D). Further validation by qMSP in a larger cohort consisting of 54 non-cirrhotic HCC livers compared to 36 samples with non-cirrhotic liver disease (Supplementary Table 2) showed higher methylation levels for all DMMs tested, except for *NKX3-2* and *GRASP* (Fig. 3E). Comparison of the DMMs by qMSP of DNA of the non-cirrhotic HCC versus 14 benign liver lesions showed comparable results: all non-cirrhotic HCC samples exhibited significant hypermethylation levels, but no statistical significance was observed for *NRIP3* and *GRASP* (Fig. 3E). At the 95 th percentile threshold of the liver control groups, pronounced hypermethylation was detected in 28 out of 54 non-cirrhotic HCC tissues for *FGF19* (51.9%), 21 for *NKX2-4* (38.9%), 40 for *SPAG6* (74.1%), 38 for *FOXD3* (70.4%), 22 for *NRIP3* (40.7%), 21 for *NKX3-2* (38.9%), 25 for *TBX4* (46.3%), 20 for *TSPYL5* (37.3%), 16 for *GRASP* (29.6%), 33 for *BOP1* (61.1%) and 23 for *C8orf82* (42.6%) (Fig. 3F). In summary, we found that these DMMs tested exhibited distinct hypermethylation in cirrhotic HCC and non-cirrhotic HCC liver tissues when compared to their respective controls.

### Tissue-derived DMMs have a limited contribution in detecting early-stage HCC in cfDNA samples from plasma (training cohort)

**Fig. 4:**
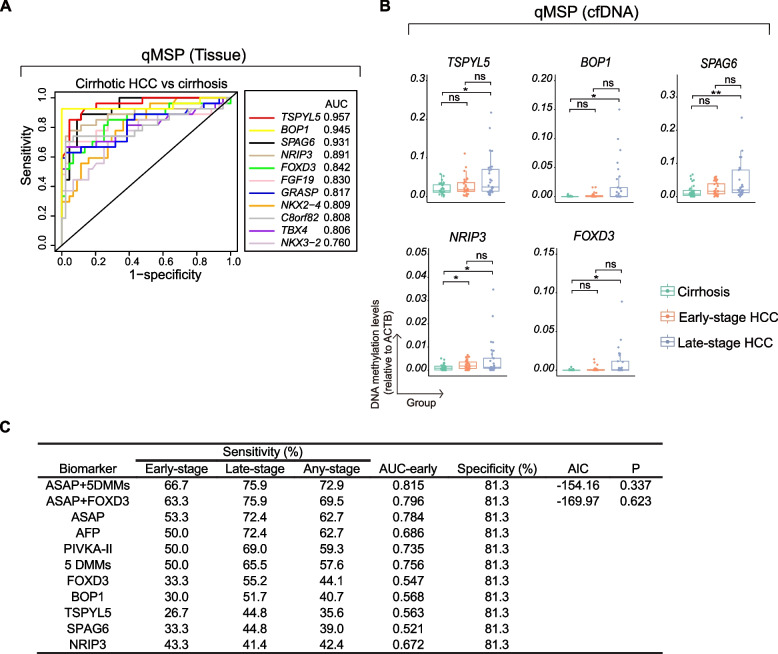
Tissue-derived DMMs had a limited contribution in detecting early-stage HCC in cfDNA (training cohort). **A** The ROC curve revealed AUC for 11 tissue-derived DMMs in distinguishing cirrhotic HCC from cirrhosis (tissue). The top 5 DMMs selected for cfDNA sample testing were *TSPYL5*, *BOP1*, *SPAG6*, *NRIP3*, and *FOXD3*. **B** qMSP-based methylation levels relative to the *ACTB* reference gene were shown for the 5 selected DMMs in 91 cfDNA samples (29 late-stage HCC, 30 early-stage HCC, and 32 cirrhosis). All 5 DMMs exhibited hypermethylation in late-stage HCC compared to cirrhosis, while only *NRIP3* showed statistical significance between early-stage HCC and cirrhosis (Mann–Whitney U test; ns, not significant; **, *p* < 0.01; *, *p* < 0.05). **C** Performance of DMMs and ASAP/GAAD in HCC detection. Binary logistical regression (Enter method) was performed to create predictive formulas for the combined 5 DMMs comprising *TSPYL5*, *BOP1*, *SPAG6*, *NRIP3*, and *FOXD3*, as well as for the combination of ASAP/GAAD and 5 DMMs. Additionally, binary logistical regression (backward elimination) was utilized to develop an alternative algorithm for the combination of ASAP/GAAD and 5 DMMs, resulting in ASAP/GAAD and *FOXD3*. The p-value for the ROC curve was compared between the combined model and the ASAP/GAAD score

To identify the DMMs with strong performance in distinguishing HCC liver tissue from cirrhosis and evaluate its potential in cfDNA, we performed ROC curve analysis based on qMSP results in liver tissue. The 11 DMMs demonstrated good ability to differentiate cirrhotic HCC from cirrhotic controls, with AUC values ranging from 0.760 to 0.957 (Fig. 4A). In contrast, only *FOXD3* and *SPAG6* maintained good performance in distinguishing non-cirrhotic HCC from non-cirrhotic control livers, with AUC values of 0.854 and 0.870, respectively (Supplementary Fig. 2). The remaining 9 DMMs showed lower AUC values for differentiating non-cirrhotic HCC from its control tissue, ranging from 0.555 to 0.800. The ultimate objective of our study was to identify a set of specific methylation markers in DNA of HCC livers that can be applied to distinguish HCC patients from control patients when cfDNA in plasma is assessed. Given that the population of interest in clinical practice is cirrhosis and that the cfDNA amount from plasma samples is limited, we selected the top-5 DMMs in liver tissues from the cirrhotic group with high AUC values ranging from 0.842 to 0.957 for further analysis: *TSPYL5*, *BOP1*, *SPAG6*, *NRIP3*, and *FOXD3* (Fig. 4A)*.*

Next, we determined whether the best performing DMMs could be used to distinguish individuals with cirrhotic HCC from cirrhotic controls by performing qMSP on cfDNA isolated exclusively from plasma. For this, we included a total of 91 patients, consisting of those with cirrhosis (*n* = 32), early-stage cirrhotic HCC (*n* = 30), and late-stage cirrhotic HCC (*n* = 29). As presented in Table 2, the gender distribution was similar between HCC patients and cirrhotic controls, while patients with cirrhosis were younger. The primary etiologies for both patient groups were ALD and MASLD. We observed that all 5 DMMs exhibited higher methylation levels in cfDNA of late-stage HCC compared to cirrhosis (*p* < 0.05). However, except *NRIP3*, no statistical significance was detected in the comparison of early-stage HCC and cirrhosis (Fig. 4B).

**Table 2:** Clinical parameters for qMSP on cfDNA samples

Training cohort (*n* = 91)				Validation cohort (*n* = 89)		
Group	Late-stage HCC	Early-stage HCC	Cirrhosis	Late-stage HCC	Early-stage HCC	Cirrhosis
N	29	30	32	24	37	28
Age, median	69	70	65	68	67	65
Gender, male	23 (79.3%)	23 (76.7%)	24 (75.0%)	18 (75.0%)	26 (70.3%)	16 (57.1%)
Cirrhosis	100%	100%	100%	100%	100%	100%
Etiology						
MASLD	10 (34.4%)	15 (50.0%)	10 (31.3%)	6 (25.0%)	17 (45.9%)	19 (67.9%)
ALD	16 (55.2%)	14 (46.7%)	18 (56.3%)	12 (50.0%)	8 (21.6%)	4 (14.3%)
HBV	0	0	0	0	0	0
HCV	0	0	0	0	0	0
Others	3 (10.3%)	1 (3.3%)	3 (9.4%)	6 (25.0%)	12 (32.4%)	5 (17.9%)
Tumor size, cm	4.8 (3.0–7.5)	2.8 (1.8–4.0)	NA	4.5 (3.5–6.0)	2.5 (2.2–3.6)	NA
BCLC Stage						
BCLC 0-A	0	30 (100%)	NA	0	37 (100%)	NA
BCLC B	15 (51.7%)	0	NA	13 (54.2%)	0	NA
BCLC C-D	14 (48.3%)	0	NA	11 (45.8%)	0	NA
AFP, ng/mL	7.7(4.6–55.2)	6.0(3.9–28.6)	4.2(3.4–5.4)	20.5(10.7–460.2)	9.0(5.5–15.2)	4.9(3.2–6.9)
PIVKA-II, mAU/ml	901(169–10,728)	196(82–971)	68(44–197)	645(345–2957)	269(107–618)	58(48–101)
ASAP/GAAD	2.35(0.21–3.68)	0.63(−0.04–2.56)	−0.47(−1.23–0.07)	1.73(1.14–5.07)	0.89(−0.07–1.51)	−0.52(−1.42–0.37)

*Abbreviations*: *HCC* hepatocellular carcinoma, *HBV* hepatitis B virus, *HCV* hepatitis C virus, *ALD* Alcohol-related liver disease, *MASLD* metabolic dysfunction-associated steatotic liver disease, *BCLC* Barcelona Clinic Liver Cancer staging system, *AFP* α-fetoprotein, *PIVKA-II* protein induced by vitamin K absence II, age, sex, AFP, and PIVKA-II, ASAP/GAAD

As shown in Fig. 4C, calculation of the DNA methylation levels resulted in poor performance of the DMMs in cfDNA with sensitivities ranging from 35.6 to 44.1% for any stage HCC, and these values were even lower for early-stage HCC (from 26.7% to 43.3% at 81.3% specificity). Also, AUCs in distinguishing early-stage HCC from cirrhosis of the individual 5 DMMs ranged from only 0.547 to 0.672. Logistic regression analysis was conducted to develop algorithms for the combination of the 5 individual DMMs. The combined 5 DMMs increased the AUC to 0.756 (50.0% sensitivity, 81.3% specificity) compared to a single DMM, which is still inferior or only marginally better than the sensitivities or AUC of AFP, PIVKA-II or the ASAP/GAAD score, recently described by our team [14]. Further analysis of the combinations of the ASAP/GAAD score with 5 DMMs or *FOXD3* (refined by backward elimination of logistical regression analysis) yielded higher AUCs of 0.815 and 0.796 versus 0.784, respectively (Fig. 4C). Although the combination of ASAP/GAAD and 5 DMMs demonstrated the highest sensitivity for early HCC detection, the AIC value (−154.16 versus −169.97) suggested that this formula's complexity is greater than that of the combination of ASAP/GAAD and *FOXD3*. Additionally, no statistically significant difference was observed when comparing the combined model to the ASAP/GAAD score alone.

For overall HCC, the performance improved for most biomarkers, with the combination of the ASAP/GAAD and 5 DMMs as well as the ASAP/GAAD and *FOXD3* achieving the highest AUC of 0.833 (72.9% sensitivity, 81.3% specificity) and 0.811 (69.5% sensitivity, 81.3% specificity), respectively, surpassing that of the ASAP/GAAD score (62.7% sensitivity, 81.3% specificity) or the 5 DMMs alone (35.6–44.1% sensitivity, 81.3% specificity). This trend was also observed among patients with late-stage HCC (Fig. 4C). In summary, we found that individual DMMs had a limited contribution in detecting early-stage HCC in cfDNA from plasma, and its additive performance benefit over the ASAP/GAAD score was only less than 15% sensitivity in our training cohort for early-stage HCC detection.

### An independent validation cohort confirms that tissue-derived DMMs only weakly contribute in detecting early-stage HCC in cfDNA samples from plasma

**Fig. 5:**
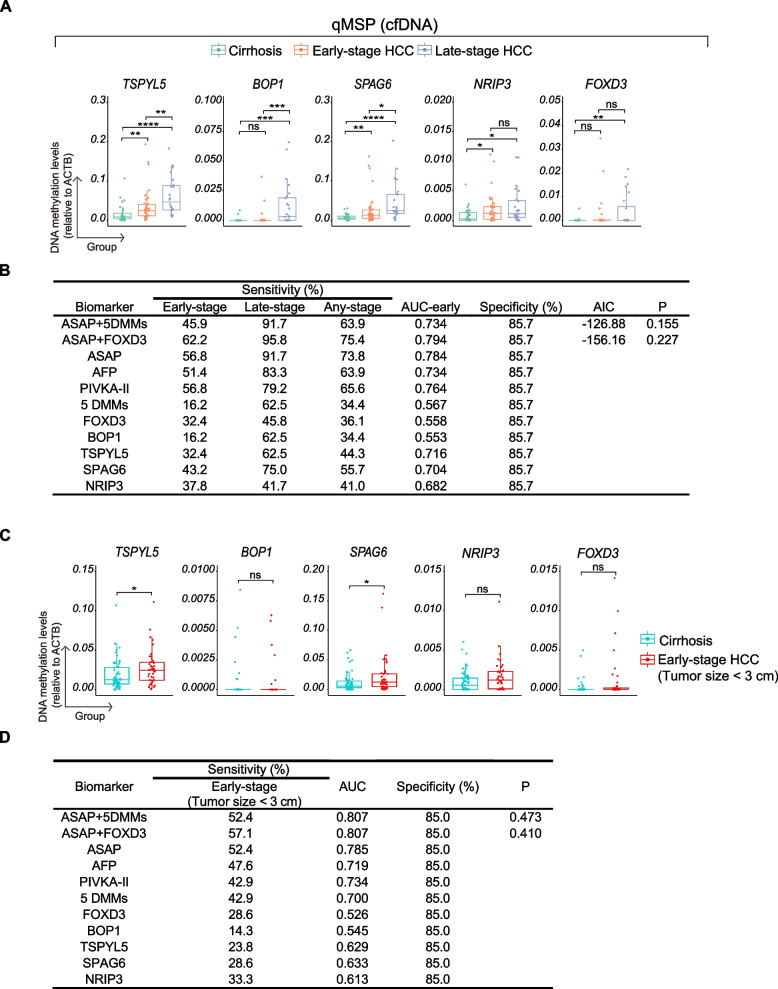
Confirmation in an independent cohort showed a limited contribution of tissue-derived DMMs in detecting early-stage HCC in cfDNA (validation cohort). **A** qMSP-based methylation levels relative to the *ACTB* reference gene were illustrated in 89 cfDNA samples (24 late-stage HCC, 37 early-stage HCC, and 28 cirrhosis). All 5 DMMs exhibited hypermethylation in cfDNA of late-stage HCC compared to cirrhosis, and *TSPYL5*, *NRIP3*, and *SPAG6* also showed statistical significance between early-stage HCC and cirrhosis (Mann–Whitney U test; ns, not significant; *, *p* < 0.05; **, *p* < 0.01; ***, *p* < 0.001; ****, *p* < 0.0001). **B** Using the model from the training cohort, the performance of the DMMs, ASAP/GAAD, and the combination of DMMs and ASAP/GAAD was evaluated for HCC detection. The p-value for the ROC curve was compared between the combined model and the ASAP/GAAD score. **C**, **D** Performance of the 5 DMMs in early-stage patients with small tumor size (< 3 cm) compared to cirrhosis, only *TSPYL5* and *SPAG6* showed statistical significance between early-stage HCC with small tumor size and cirrhosis (Mann–Whitney U test; ns, not significant; * *p* < 0.05)

To confirm our results on the performance of the DMMs panel on cfDNA in an independent validation cohort, qMSP was performed on DNA from late-stage cirrhotic HCC patients (*n* = 24), early-stage cirrhotic HCC patients (*n* = 37), and cirrhosis patients (*n* = 28). In line with the findings in our discovery cohort, all 5 DMMs exhibited hypermethylation in cfDNA of late-stage cirrhotic HCC compared to cirrhosis (*p* < 0.05; Fig. 5A). Notably, *TSPYL5*, *SPAG6*, and *NRIP3* also demonstrated hypermethylation in early-stage cirrhotic HCC in comparison to their cirrhotic counterpart (*p* < 0.05).

Although some of the 5 DMMs showed statistical significance in comparisons to qMSP data on cfDNA of early-stage cirrhotic HCC and cirrhosis, their performance in early HCC detection was still suboptimal, with sensitivities ranging from 16.2% to 43.2% at 85.7% specificity (Fig. 5B). In late-stage HCC, sensitivity increased for all biomarkers, but the performance was still inferior to AFP, PIVKA-II or the ASAP/GAAD score (Fig. 5B).

Next, we calculated the AUC for the 5 DMMs and the ASAP/GAAD score in early HCC detection (Fig. 5B). Using the same model from the training cohort, the combined 5 DMMs showed a suboptimal performance in validation cohort, with AUC 0.567. The combination of the 5 DMMs and the ASAP/GAAD score demonstrated poorer performance in both AUC (0.734 versus 0.794) and AIC (−126.88 versus −156.16) than the combination of ASAP/GAAD and *FOXD3* for early-stage HCC detection. This suggests that the AUC of ASAP/GAAD and 5 DMMs in the training cohort may have been overestimated. In the validation cohort, the combination of ASAP/GAAD and *FOXD3* (62.2% sensitivity at 85.7% specificity) demonstrated better performance than ASAP/GAAD score alone (56.8% sensitivity at 85.7% specificity) in early HCC detection; however, no statistical significance was observed. The sensitivity also increased in late-stage HCC, with the combination of ASAP/GAAD and *FOXD3* showing the highest sensitivity at 95.8% with 85.7% specificity, which is slightly higher than that of the ASAP/GAAD score (91.7% sensitivity at 85.7% specificity).

Additional sub-analysis of the DMMs qMSP results on cfDNA from 42 early-stage cirrhotic HCC patients with small tumor sizes (< 3 cm) in both the training and validation cohort showed comparable results as compared to entire early-stage cohorts (Fig. 5C). Again, relatively low sensitivities and performances were observed for the individual DMMs in cfDNA between early-stage HCC patients with small tumor sizes and those with cirrhosis, and the combination of ASAP/GAAD with the 5 DMMs or with *FOXD3* achieved an AUC of 0.807 and 0.807 versus 0.785 for ASAP/GAAD alone (Fig. 5D). In summary, we found that the performance of individual DMMs is limited in early-stage HCC detection. The combination of ASAP/GAAD and the DMMs demonstrated only marginally higher sensitivity compared to ASAP/GAAD alone in the validation cohort for early-stage HCC.

To address potential tissue–plasma discordance caused by inter-patient variations, we evaluated DNA methylation changes on 9 paired tissue–plasma samples from the same cirrhotic HCC patients using qMSP for the selected 5 DMMs (Supplementary Table 3). As presented in Supplementary Fig. 3, DNA methylation levels were substantially lower in cfDNA compared to liver tissues, indicating a low contribution of tumor-derived DNA in the blood samples.

### By MeD-seq analysis, DNA methylation patterns of cfDNA are similar between cirrhotic HCC and cirrhosis, but distinct from those of healthy individuals

**Fig. 6:**
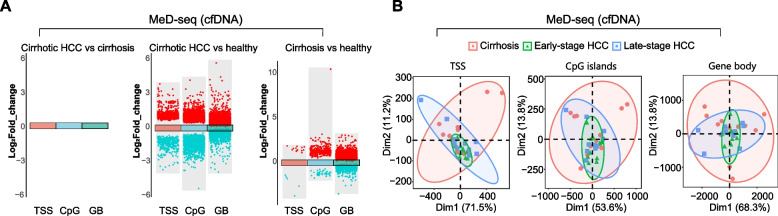
By MeD-seq analysis, DNA methylation patterns of cfDNA are similar between cirrhotic HCC and cirrhosis, but distinct from those of healthy individuals. **A** MeD-seq was conducted on samples from patients with ALD-related cirrhotic HCC (*n* = 20), ALD-related cirrhosis (*n* = 10), and healthy individuals (*n* = 10). No DMRs were observed when comparing cirrhotic HCC and cirrhosis. However, distinct DNA methylation patterns were identified in both cirrhotic HCC and cirrhosis when compared to healthy individuals. **B** PCA analysis was conducted using cirrhotic HCC tissue-specific DMMs, with a total of 3842, 4417, and 5154 DMRs selected for this analysis in the transcription start sites (TSS), CpG islands (CpG), and gene bodies (GB), respectively. DNA methylation patterns were similar among cirrhosis (*n* = 12), early-stage cirrhotic HCC (*n* = 10), and late-stage cirrhotic HCC (*n *= 10). DMRs located on the X- and Y-chromosome were removed to avoid gender-related effects

Given the poor performance of tissue-derived DMMs using cfDNA qMSP on the training and the validation cohort, we decided to further confirm the findings using the unbiased MeD-seq sequencing approach. One of the advantages of MeD-seq over methods involving bisulfite conversion is its suitability for high-throughput DNA methylation changes in small quantities of cfDNA [30]. MeD-seq on cfDNA was performed on samples from patients with ALD, consisting of late-stage cirrhotic HCC (*n* = 10), early-stage cirrhotic HCC (*n* = 10), and cirrhotic controls (*n* = 12), along with 10 healthy individuals (Table 1). To our surprise, no DMRs were found in cirrhotic HCC (*n* = 20) compared to cirrhotic controls (Fig. 6A and Supplementary Fig. 4). However, patients with cirrhotic HCC and cirrhosis displayed distinct methylation patterns and increased hypermethylation when compared to healthy individuals (Fig. 6A). Subsequently, PCA was conducted to compare the DNA methylation patterns for cfDNA samples based on the previous cirrhotic HCC tissue-specific DMMs. A total of 3842, 4417, and 5154 DMRs were selected for this analysis in the TSS, CpG islands, and gene bodies, respectively. As shown in Fig. 6B, the methylation patterns in cfDNA could not be distinctly separated among cirrhosis, early-stage cirrhotic HCC, and late-stage cirrhotic HCC. Furthermore, the variations and extensive DNA methylation changes associated with cirrhosis in cfDNA largely overlapped with the methylation patterns characteristic for early-stage HCC and late-stage HCC.

## Discussion

In this study, we conducted bimodal tissue-blood genome-wide DNA methylation sequencing by MeD-seq on samples from patients with HCC to evaluate the specificity of DMMs in early HCC detection. In liver tissues, distinct DNA methylation patterns were identified between HCC cases and controls. However, in blood samples, the selected DMMs demonstrated limited sensitivity for early HCC detection. Further validation using MeD-seq showed no DMRs between cirrhotic HCC and cirrhosis in cfDNA, while elevated DNA methylation levels were observed in both of these groups when compared to healthy controls.

Despite strong performance in distinguishing cirrhotic HCC from cirrhotic liver tissues, we observed relatively low performance of tumor-derived DMMs in cfDNA in both the training cohort and the validation cohort. In fact, evaluation of individual DMMs resulted in a sensitivity of less than 45%, and the combination of 5 DMMs resulted in a sensitivity of 50%. The combined ASAP/GAAD score with DMMs resulted in a sensitivity of around 65% in distinguishing early-stage cirrhotic HCC from cirrhosis in cfDNA, which was only less than 15% higher than the values for the ASAP/GAAD score alone. These findings clearly demonstrate that it challenging to distinguish early-stage cirrhotic HCC from cirrhosis using DMMs on cfDNA. This difficulty may be related to tumor size, tumor heterogeneity, and the challenge of extracting sufficient circulating tumor DNA from the substantial background of cfDNA from other sources in blood of patients with underlying cirrhosis. cfDNA is shed into the circulation by cells undergoing apoptosis or necrosis [33]. In healthy individuals, only 1–3% of cfDNA originates from hepatocytes, while the majority comes from other cell types, such as leukocytes and erythrocyte progenitors [20, 34]. This complicates the evaluation of liver-specific cfDNA from blood samples. Furthermore, in early-stage cirrhotic HCC, the tumor represents only a small portion of the liver, with the remaining liver tissue predominantly affected by cirrhosis, thus making the detection of liver tumor-specific DNA in blood even more challenging. Several studies are in line with our observations and have also reported on the low sensitivity of cfDNA-based biomarkers in detecting early-stage HCC with sensitivities up to 60% depending on the subgroup analysis [35–38], as well as on a significant increase of DNA methylation levels in large HCC tumors [27].

In our cfDNA analyses using MeD-seq, we observed increased DNA methylation levels in cfDNA from patients with cirrhosis compared to healthy individuals. This finding suggests that these methylation changes occur prior to HCC development and are associated with disease progression. Indeed, a number of studies have reported aberrant methylation events occurring before carcinogenesis. In those tissues, DNA methylation changes were associated with liver cirrhosis [26], pulmonary fibrosis [39], and atherosclerosis [40]. In cfDNA, DMMs could also be used to distinguish liver cirrhosis [29], advanced colorectal adenomas [41], and fibrotic interstitial lung diseases [42] from healthy individuals. Our observation from the MeD-seq analysis that cfDNA cirrhotic patients have altered DNA methylation as compared to healthy control individuals is relevant, and warrants concern on the appropriate patient control groups that need to be selected as comparison. In previous studies on DMMs in HCC that reported excellent performance in HCC detection, the control groups included healthy individuals [43, 44], non-cirrhotic controls or cases with an unknown cirrhosis status [45–47]. In two studies with over 85% cirrhotic controls, the combination of DMMs with either AFP and AFP-L3 or AFP and gender improved sensitivity for early-stage HCC detection by 14–15% in the primary cohort, compared to the GALAD score alone [27, 28]. However, in the validation cohort, the sensitivity increase was only 4% compared to the GALAD score alone [28]. Besides the effects of the cirrhotic background on DNA methylation profiles, in clinical practice, patients with cirrhosis are considered a high-risk population and should undergo HCC surveillance whereas healthy individuals rarely progress to HCC. Therefore, including healthy individuals in cirrhotic HCC studies may not effectively fulfill the objective of identifying early biomarkers for HCC detection. Additionally, we attempted to track DNA methylation signals using hepatocyte-specific markers, such as albumin (ALB) and apolipoprotein A-II (APOA2); however, we did not observe methylation in these two genes in cfDNA (data not shown). Furthermore, we examined hepatocyte-specific CpGs identified in a previous study [20] and found hypermethylation of these CpGs only in cirrhotic HCC liver tissues, while hypermethylation could not be convincingly demonstrated in HCC cfDNA (Supplementary Fig. 5). However, differences between the platforms used (Illumina versus MeD-seq) may warrant caution in drawing definitive conclusions.

In our previous study [17], we tested the DNA methylation levels of 4 previously reported tissue-derived DMMs, *HOXA1*, *CLEC11 A*, *AK055957*, and *TSPYL5* [48], on both non-cirrhotic and cirrhotic HCC tissue. We observed higher AUC values for some of the DMMs (i.e. *TSPYL5*) in cirrhotic HCC compared to non-cirrhotic HCC (tissue), which is consistent with the data in the current study. Although *HOXA1* has been reported as a reliable DMM for early HCC detection in cfDNA [27, 28], we did not identify a specific hypermethylated site for *HOXA1* through our genome-wide DMRs analysis of MeD-seq data in the comparison of cirrhotic HCC and cirrhosis tissue. This may be related to tumor heterogeneity and the etiology of the samples, as we specifically selected ALD-related liver disease for MeD-seq in this study.

In order to identify the most optimal biomarker for detection of early-stage HCC, an effective strategy previously used is a combination of DMMs with other types of biomarkers, such as AFP [27, 28]. In our current study we demonstrated that the diagnostic power for early HCC detection in cfDNA was weakly enhanced by integrating multi-analyte detection methods, i.e. the ASAP score and DMMs. However, this approach increases the complexity and cost of the test, raising concerns about its cost-effectiveness.

Our study has several limitations. Firstly, we did not assess DNA methylation changes in a longitudinal cohort, which makes that we cannot conclude on dynamic changes in cfDNA methylation over time. Secondly, since HCC is a highly heterogeneous disease, we limited our DMMs analysis of cfDNA to samples with non-viral etiology, and therefore comparison with samples from patients with a viral etiology cannot be made. Despite these limitations, our study provides valuable insights into methylation patterns in liver tissue and blood, emphasizing the limited role of cfDNA in early HCC detection compared to cirrhosis patients when using current methods.

## Conclusion

In summary, our findings indicate that the performance of DNA methylation in cfDNA was modest in differentiating early-stage cirrhotic HCC from cirrhotic controls. This is partly due to the dilution of tumor-related methylation levels by cfDNA from other cell types and the DNA methylation changes in the blood of cirrhotic patients compared to healthy individuals. This raises questions about the effectiveness of DNA methylation markers as non-invasive biomarkers for early-stage cirrhotic HCC detection.

## Supplementary Information

Supplementary Material 1

## Data Availability

The sequencing data reported in this paper are deposited in NCBI Sequence Read Archive (SRA) database with accession numbers PRJNA1195705 and PRJNA1147893. Any additional information required to reanalyze the data reported in this paper is available from the corresponding author upon request.
