# Supplementary material for: Genome-Wide Methylation Sequencing to Identify DNA Methylation Markers for Early-stage Hepatocellular Carcinoma in Liver and Blood
Source: J Exp Clin Cancer Res. 2025 May 15;44:144. doi: 10.1186/s13046-025-03412-9 (PMC12079860; doi:10.1186/s13046-025-03412-9)
Supplement: Supplementary file 1 — Supplementary Material 1 [file 13046_2025_3412_MOESM1_ESM.docx]

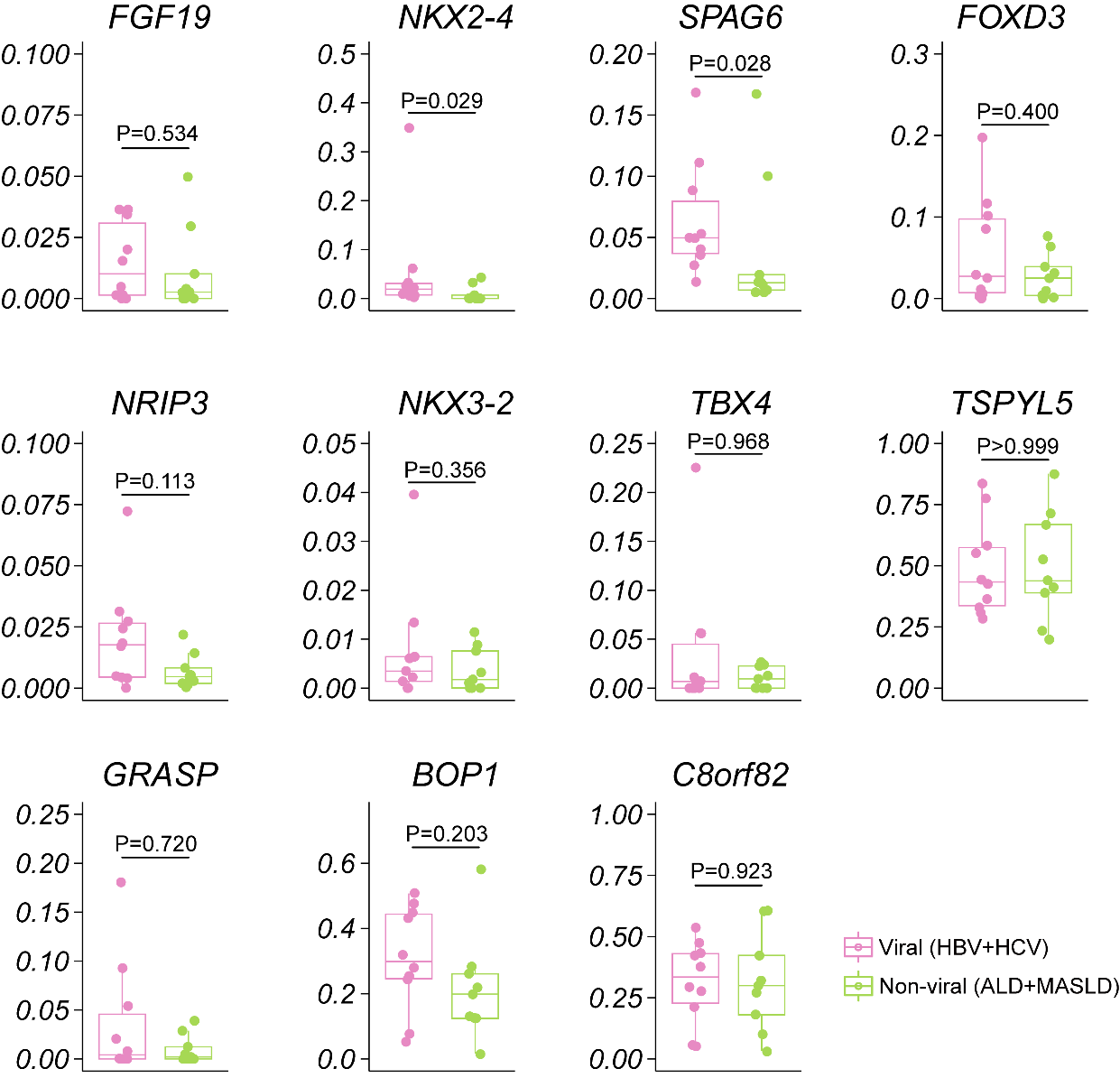


**Supplementary Figure 1. Comparison of etiology between viral and non-viral-related cirrhotic HCC tissues using qMSP for the 11 DMMs.** qMSP-based DNA methylation levels, normalized by *ACTB*, were assessed in 10 viral (HBV+HCV) and 9 non-viral-related (ALD+MASLD) cirrhotic HCC samples for the 11 DMMs. The Mann-Whitney U test was used to calculate p-values.


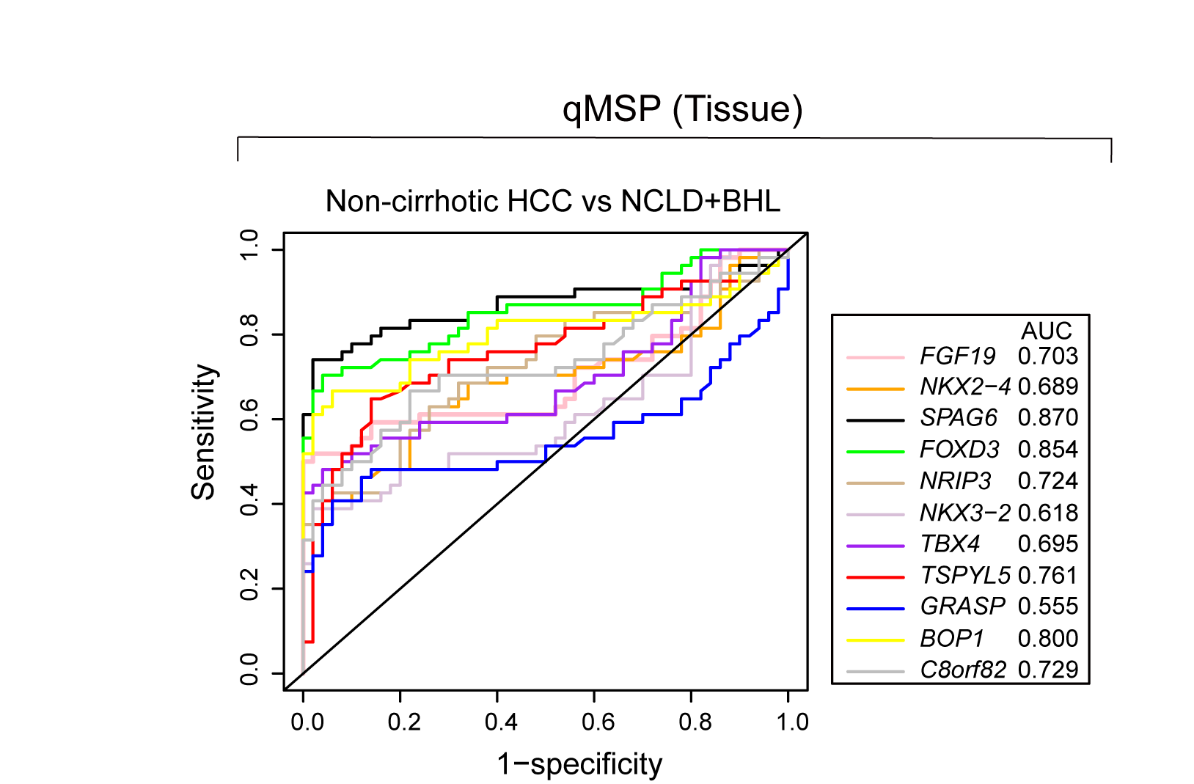


**Supplementary Figure 2. Performance of tissue-derived DMMs in distinguishing non-cirrhotic HCC from non-cirrhotic controls.** qMSP-based DNA methylation levels normalized by ACTB were assessed in 54 non-cirrhotic HCC and 50 non-cirrhotic controls tissues (36 non-cirrhotic liver disease samples and 14 benign hepatic lesions). The AUC was calculated for the selected 11 DMMs in the comparison between non-cirrhotic HCC and non-cirrhotic controls.


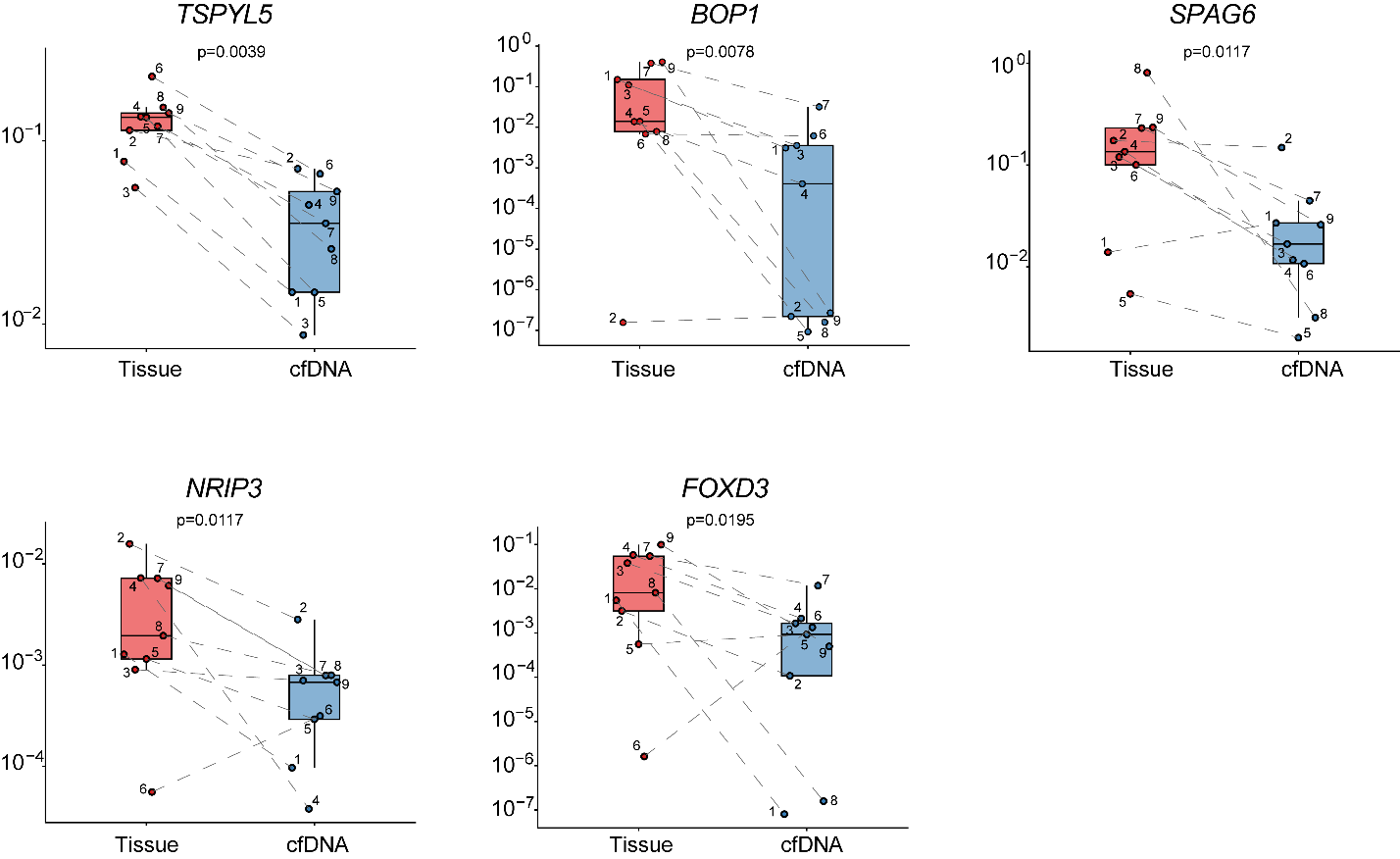


**Supplementary Figure 3. Comparison of DNA methylation levels in paired tissue and cfDNA samples.** Quantitative methylation-specific PCR (qMSP) was performed on 9 paired liver tissue and plasma cfDNA samples from cirrhotic HCC patients, targeting five selected DMMs. DNA methylation levels were normalized to *ACTB*. Statistical differences between paired tissue and cfDNA samples were assessed using the Wilcoxon signed-rank test.


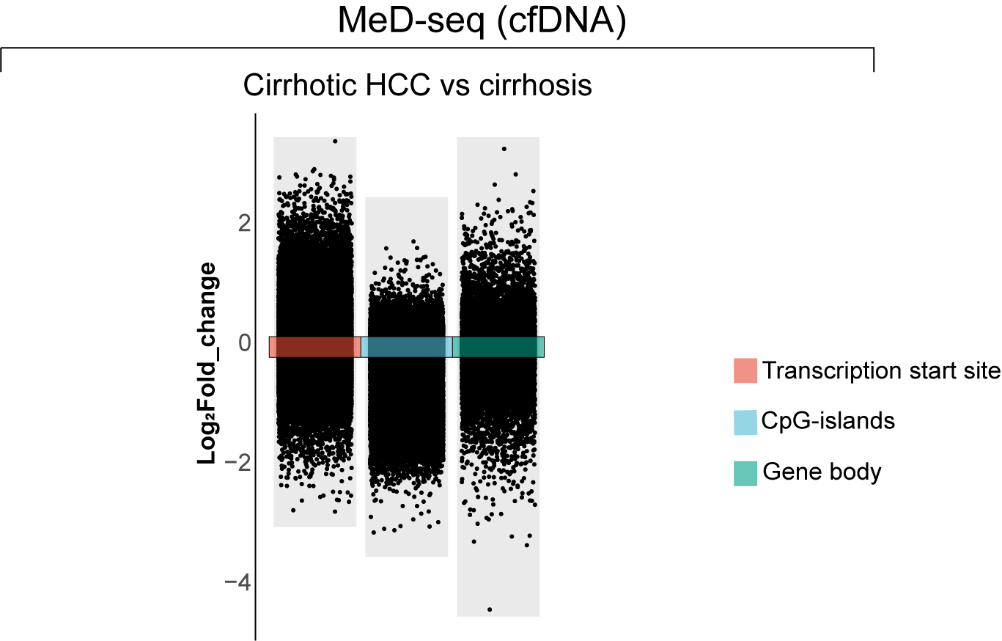


**Supplementary Figure 4. MeD-seq analysis of cfDNA from cirrhotic HCC and cirrhosis with ALD-related liver disease.** MeD-seq was conducted on cfDNA from 20 cirrhotic HCC patients and 10 cirrhosis patients with ALD-related liver disease. DESeq2 was used to calculate the DMRs on TSS, CpG islands, and gene bodies between cirrhotic HCC and cirrhosis. No DMRs were identified in this comparison, with black dots indicating no statistically significant genes (FDR ≥ 0.01). DMRs located on the X- and Y-chromosome were removed to avoid gender-related effects.

**
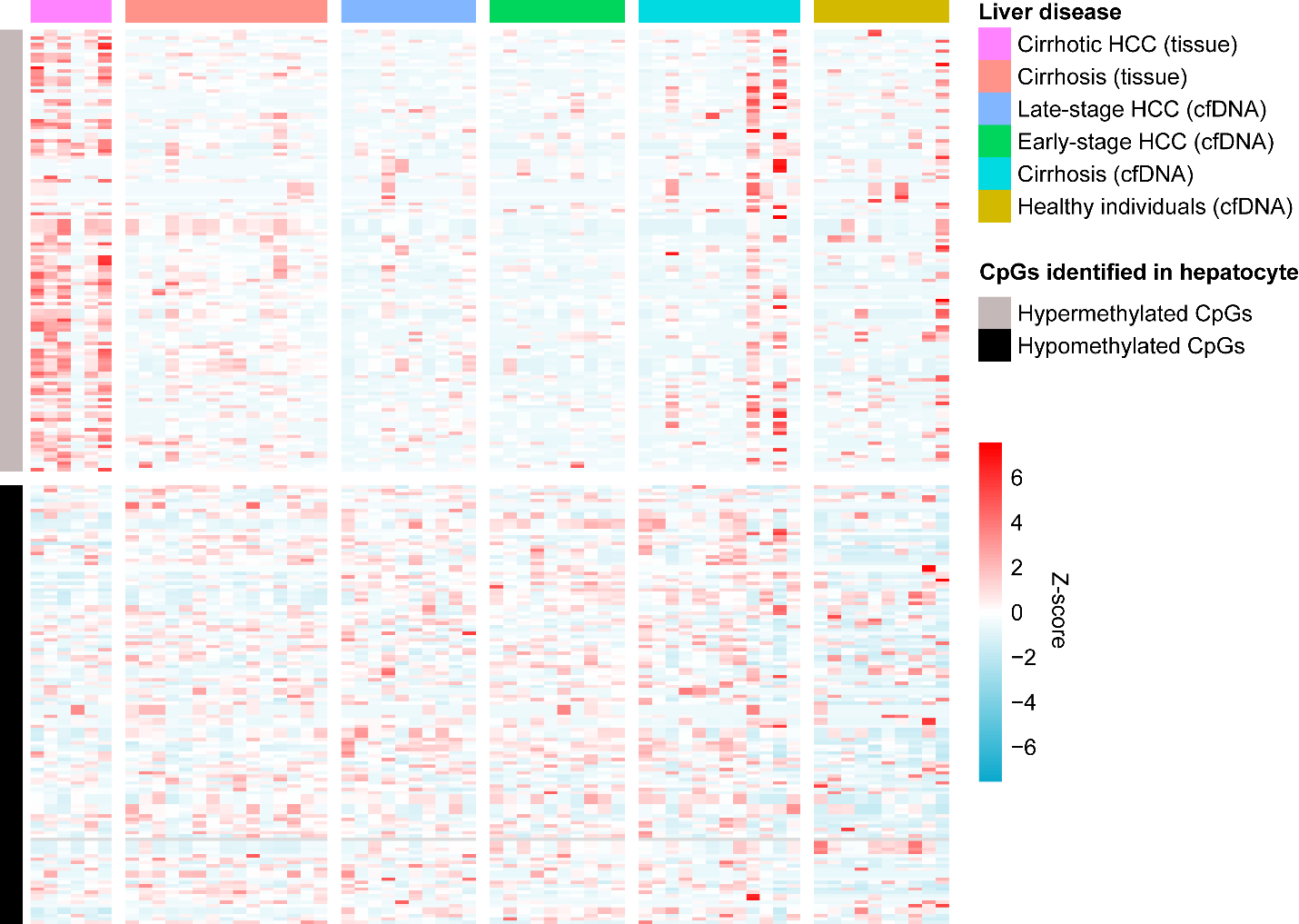
**

**Supplementary Figure 5. Hepatocyte-specific CpGs in liver tissues and cfDNA.** A previous study (Moss et al., *Nature Communications*, 2018) identified hepatocyte-specific CpGs by comparing DNA methylation levels in hepatocytes to those in 24 other cell types. A list of 133 hypermethylated and 132 hypomethylated hepatocyte-specific CpGs were evaluated for the corresponding DNA methylation score by MeD-seq analysis of our cohorts of liver tissue and cfDNA. Z-scores are presented in the heatmap.

**Supplementary Table 1. DNA methylation-specific primer and probe sequences**

| Gene | Primer | Sequence 5’ to 3’ | Amplicon length (bp) | Annealing temperature (°C) |
| --- | --- | --- | --- | --- |
| *FGF19* | Forward primer | GTTCGGTTACGGTTAGTTAGAGGTC | 81 | 62 |
|  | Reverse primer | CCCAAAAAAATACCATACGAAACG |  |  |
|  | Probe | GTTAGGATTTATACGTGGATTAT-**MGB*** |  |  |
| *NKX2-4* | Forward primer | TATTTGACGAGTAGCGTGGGTTC | 87 | 62 |
|  | Reverse primer | AACTACCCGCCTACACGAACG |  |  |
|  | Probe | CGTCGTATTAGTCGGTGGT-**MGB*** |  |  |
| *SPAG6* | Forward primer | GGTGGGTTTTATCGATTTTTTTTTTC | 84 | 62 |
|  | Reverse primer | ACTCCGCCACCATCTACACG |  |  |
|  | Probe | CGAGTAATATTAGAAGGTTAGGATT-**MGB*** |  |  |
| *FOXD3* | Forward primer | TTCGTTGTTTTTTTTTCGTCGTC | 95 | 62 |
|  | Reverse primer | ACACCGTCTAACCGAACATATCG |  |  |
|  | Probe | TTTTTTTCGGCGGCGGTAGCGTT |  |  |
| *NRIP3* | Forward primer | TGAGGTCGTTTAGGGGTAGTAGGTC | 90 | 62 |
|  | Reverse primer | CGACGTCACTACGACAACAACG |  |  |
|  | Probe | CGGAGTTTTTGTGGATGAATTGTATCGTTTGT |  |  |
| *NKX3-2* | Forward primer | GTAGTTGTTTTGGTATCGGTAGGAGAC | 109 | 62 |
|  | Reverse primer | CGACTCCCGCTATCTACTATTAACG |  |  |
|  | Probe | TAGTAGAGAGTTTTCGGCGTTT-**MGB*** |  |  |
| *TBX4* | Forward primer | GGTCGGGGATTCGTAGAAGTC | 126 | 62 |
|  | Reverse primer | AAACTCGACAACCAAAAAAACTCG |  |  |
|  | Probe | TTAGATTCGGTCGTGGAGC-**MGB*** |  |  |
| *TSPYL5* | Forward primer | TTTAGGGGGAGTCGGTAGGC | 81 | 60 |
|  | Reverse primer | GAAATAAAATAACCTAAAAACCGCTACG |  |  |
|  | Probe | TTTTTTTTTCGAGTCGGAGGAGTTGCG |  |  |
| *GRASP* | Forward primer | CGGATTTTCGATTCGGAAGTC | 86 | 62 |
|  | Reverse primer | GCTAAAAACCCAAAAATAACGACTACG |  |  |
|  | Probe | CGGTTTCGATTTCGGGAT-**MGB*** |  |  |
| *BOP1* | Forward primer | CGTAGGAGGCGGTATGAGTAGC | 120 | 60 |
|  | Reverse primer | GAACTCACCTCGAAATACAAATAACG |  |  |
|  | Probe | TTCGTTACGTTGCGTTCGGCGTC |  |  |
| *C8orf82* | Forward primer | TCGGAGCGTATTAGGGTTAGAGTC | 100 | 60 |
|  | Reverse primer | GCGAAAACGTCAAATAAAAAAACG |  |  |
|  | Probe | CGTTTGGTGATTTTGGGTC-**MGB*** |  |  |
| *ACTB* | Forward primer | AACCAATAAAACCTACTCCTCCCTTAA | 133 | 62 |
|  | Reverse primer | TGGTGATGGAGGAGGTTTAGTAAGT |  |  |
|  | Probe | ACCACCACCCAACACACAATAACAAACACA |  |  |

Abbreviations: MGB*, minor groove binder; bp, base pairs; NK2 homeobox 4, NKX2-4; nuclear receptor interacting protein 3, NRIP3; T-box transcription factor 4, TBX4; NK3 homeobox 2, NKX3-2; sperm-associated antigen 6, SPAG6; fibroblast growth factor 19, FGF19; testis-specific protein Y-encoded-like 5, TSPYL5; forkhead box D3, FOXD3; grp1 (general receptor for phosphoinositides-1)-associated scaffold protein, GRASP; block of proliferation 1, BOP1; chromosome 8 Open Reading Frame 82, C8orf82; β-actin, ACTB.

**Supplementary Table 2. Clinical Characteristics of Study Participants for qMSP on liver tissues**

| Sample | Liver tissue(n=175) | | | | |
| --- | --- | --- | --- | --- | --- |
| Group | Cirrhotic HCC | Cirrhosis | Non-cirrhotic HCC | Non-cirrhotic live disease | Benign hepatic lesions |
| N | 27 | 44 | 54 | 36 | 14 |
| Age, median | 72 | 62 | 65 | 52 | 41 |
| Gender, male | 20 (74.1%) | 33 (75.0%) | 29 (53.7%) | 25 (69.4%) | 2(14.3%) |
| Cirrhosis | 100% | 100% | 0% | 0% | 0% |
| Etiology |  |  |  |  |  |
| MASLD | 3 (11.1%) | 1 (2.3%) | 15 (27.8%) | 4 (11.1%) | 0 |
| ALD | 6 (22.2%) | 19 (43.2%) | 8 (14.8%) | 13 (36.1%) | 0 |
| HBV | 5 (18.5%) | 10 (22.7%) | 4 (7.4%) | 4 (11.1%) | 0 |
| HCV | 5 (18.5%) | 4 (9.1%) | 1 (1.9%) | 3 (8.3%) | 0 |
| Cryptogenic | 2 (7.4%) | 6 (13.6%) | 26 (48.1%) | 12 (33.3%) | 0 |
| HCA | 0 | 0 | 0 | 0 | 11 (78.6%) |
| FNH | 0 | 0 | 0 | 0 | 3 (21.4%) |
| Others | 6 (22.2%) | 4 (9.1%) | 0 | 0 | 0 |
| Stages |  |  |  |  |  |
| Early-stage/  BCLC 0-A | 18 (66.7%) | NA | 25 (46.3%) | NA | NA |
| Intermediate/  BCLC B | 7 (25.9%) | NA | 26 (48.1%) | NA | NA |
| Advanced/  BCLC C-D | 2 (7.4%) | NA | 3 (5.6%) | NA | NA |

Abbreviations: Hepatocellular carcinoma, HCC; hepatitis B virus, HBV; hepatitis C virus, HCV; alcohol-related liver disease, ALD; metabolic dysfunction-associated steatotic liver disease, MASLD; focal nodular hyperplasia, FNH; hepatocellular adenoma, HCA; not available, NA.

**Supplementary Table 3. Clinical characteristics of cirrhotic HCC patients with paired tissue and plasma samples.**

| Patient ID | Age | Gender | Etiology | Largest tumor size, cm |
| --- | --- | --- | --- | --- |
| 1 | 59 | Female | HCV | 1.8 |
| 2 | 58 | Male | Cryptogenic | 14.1 |
| 3 | 64 | Female | ALD | 8 |
| 4 | 73 | Male | MASLD | 2.5 |
| 5 | 86 | Male | Cryptogenic | 11.5 |
| 6 | 61 | Male | ALD | 4 |
| 7 | 61 | Male | HCV | 7.7 |
| 8 | 52 | Male | HBV | 6.2 |
| 9 | 72 | Male | ALD | 3.7 |

Abbreviations: Hepatocellular carcinoma, HCC; hepatitis B virus, HBV; hepatitis C virus, HCV; alcohol-related liver disease, ALD; metabolic dysfunction-associated steatotic liver disease, MASLD.
